# Aspects that influence the self-care of patients living with human immunodeficiency virus^*^

**DOI:** 10.1590/1518-8345.2746.3112

**Published:** 2019-03-18

**Authors:** Rúbia Aguiar Alencar, Ana Beatriz Henrique Parenti, Camila de Carvalho Lopes, Fabiana Tomé Ramos, Suely Itsuko Ciosak

**Affiliations:** 1 Universidade Estadual Paulista, Faculdade de Medicina de Botucatu, Botucatu, SP, Brazil.; 2 Universidade de São Paulo, Escola de Enfermagem, São Paulo, SP, Brazil.

**Keywords:** Self Care, Acquired Immunodeficiency Syndrome, HIV, Nursing Care, Adult Health, Ambulatory Care, Autocuidado, Síndrome da Imunodeficiência Adquirida, HIV, Cuidados de Enfermagem, Saúde do Adulto, Assistência Ambulatorial, Autocuidado, Síndrome de Inmunodeficiencia Adquirida, VIH, Atención de Enfermería, Salud del Adulto, Atención Ambulatoria

## Abstract

**Objective:**

to analyze aspects related to the increase or decrease of self-care in patients living with human immunodeficiency virus treated in a specialized outpatient service.

**Method:**

analytical cross-sectional study with 135 patients aged 18 and over, of both sexes, who are followed up on the service. The independent variables and outcomes were collected from the nursing consultation instrument, whose theoretical reference is the Orem’s Theory. The data were analyzed by parametric approach. Relationships or differences were considered significant if p <0.05. The analysis was done with SPSS v21.0 software.

**Results:**

most participants were male (56.3%), with a mean age of 42.1 years. Patients who needed to conceal the diagnosis had less self-care (β = -0.72 (-1.38, -0.06), p <0.031). The chance of performing self-care decreased with increasing age (OR = 0.93 (0.89, 0.97), p <0.003). On the other hand, patients with a permanent partner had a higher chance of performing self-care (OR = 3.46 (1.27, 9.46), p <0.015).

**Conclusion:**

aspects related to the increase or decrease of self-care in patients living with human immunodeficiency virus were evidenced. However, further studies are necessary to emphasize the analytical character of the self-care of these patients.

## RESUMO

**Objetivo:**

analisar aspectos relacionados ao aumento ou diminuição do autocuidado nos pacientes vivendo com vírus da imunodeficiência humana atendidos em serviço de ambulatório especializado.

**Método:**

estudo transversal de caráter analítico com 135 pacientes com idade maior ou igual a 18 anos, de ambos os sexos, que fazem acompanhamento no serviço. As variáveis independentes e os desfechos foram coletados do instrumento da consulta de enfermagem, que tem como referencial teórico a Teoria de Orem. Os dados foram analisados por abordagem paramétrica. Relações ou diferenças foram consideradas significativas se p < 0,05. A análise foi feita com o *software* SPSS v21.0.

**Resultados:**

maioria do sexo masculino (56,3%), com média de idade de 42,1 anos. Os pacientes que necessitaram ocultar o diagnóstico desempenharam menos autocuidado (β= -0,72 (-1,38; -0,06); p<0,031). A chance de realizar o autocuidado diminuiu em relação ao aumento da idade (OR=0,93 (0,89;0,97);p<0,003). Por outro lado, encontrou-se que o paciente que tem parceria fixa possui maior chance de desempenhar o autocuidado (OR=3,46 (1,27;9,46); p<0,015).

**Conclusão:**

foram evidenciados aspectos relacionados ao aumento ou à diminuição do autocuidado nos pacientes que vivem com vírus da imunodeficiência humana. No entanto, fazem-se necessários outros estudos que privilegiem o caráter analítico do desempenho do autocuidado desses pacientes.

## RESUMEN

**Objetivo:**

analizar los aspectos relacionados al aumento o disminución del autocuidado en los pacientes viviendo con el virus de la inmunodeficiencia humana que fueron atendidos en servicio de ambulatorio especializado.

**Método:**

estudio transversal de carácter analítico con 135 pacientes con edad mayor o igual a 18 años, de ambos sexos, que hacen seguimiento en el servicio. Las variables independientes y los resultados fueron recolectados del instrumento de la consulta de enfermería, que tiene como referencial teórico la Teoría de Orem. Los datos se analizaron mediante un enfoque paramétrico. Las relaciones o diferencias se consideraron como significativas si p <0,05. El análisis se realizó con el software SPSS v21.0.

**Resultados:**

la mayoría del sexo masculino (56,3%), con una media de edad de 42,1 años. Los pacientes que necesitaban ocultar el diagnóstico desempeñaron menos autocuidado (β= -0,72 (-1,38; -0,06), p<0,031). La probabilidad de realizar el autocuidado disminuyó en relación al aumento de la edad (OR=0,93 (0,89, 0,97), p<0,003). Por otro lado, se encontró que el paciente que tiene asociación fija tiene mayor probabilidad de desempeñar el autocuidado (OR=3,46 (1,27, 9,46), p<0,015).

**Conclusión:**

se evidenciaron aspectos relacionados al aumento o a la disminución del autocuidado en los pacientes que viven con virus de la inmunodeficiencia humana. Sin embargo, se hacen necesarios otros estudios que privilegien el carácter analítico del desempeño del autocuidado de esos pacientes.

## Introduction

Since the beginning of the Acquired Immunodeficiency Syndrome (AIDS) epidemic, many researchers from all over the world have been conducting research to answer questions about this disease^1^ ^-^ ^5^. 

However, even after nearly 40 years of the discovery of the Human Immunodeficiency Virus (HIV), there is still a deficit in the care provided to people living with HIV/AIDS (PLWHA), especially when it comes to living and coping with the infection, in the quest to encourage the patients’ autonomy to perform their self-care and also in the care of their psychosocial needs. In this way, providing care for chronic diseases like AIDS is a growing challenge in the world^1^. It is believed that, to meet the challenges of caring for people with chronic diseases, it is necessary to identify factors that may influence people to become more involved in self-care.

In this perspective, the importance of the Nursing Process is emphasized as a methodological tool that guides professional nursing care and that must be developed in an intentional way^6^. In this way, this tool can contribute to reduce the gap that still exists in the assistance to PLWHA^2^ ^,^ ^7^ ^-^ ^9^. 

In institutions providing outpatient health services, the Nursing Process corresponds to the Nursing Consultation^2^, which makes it possible to identify the specific needs of PLWHA, to promote quality of life and to encourage them to perform self-care.

The Federal Nursing Council (COFEN) Resolution 358/2009 provides for the Systematization of Nursing Care and makes the Nursing Consultation mandatory in the development of nursing care at all levels of health care, whether in a public or private institution, making possible the operationalization of the Nursing Process. However, the nurse must have the understanding and the clear decision of the use of the Nursing Process, and COFEN Resolution 358/2009 should not be the main motivation for the use of this tool.

Considering the commitment to assist the individual living with HIV/AIDS, the nurse, through the Nursing Consultation, which must be based on a theoretical framework, can diagnose the patient’s needs, proceed with the prescription of care and then evaluate the interventions along with patient, having the opportunity to develop a work aimed at improving the quality of life of these subjects^2^.

We chose to use Dorothea Orem’s General Theory, also known as the Self-Care Deficit Nursing Theory, composed of three interrelated theories: Self-Care Theory, Self-Care Deficit Theory, and Nursing Systems Theory^10^. In the present study, the Self-Care Theory was used to identify self-care deficits and to observe the extent to which the patient is able to perform self-care, which is fundamental for maintaining the health of the individual living with HIV/AIDS^7^ ^-^ ^9^. This theory has three requisites for self-care: Universal Self-Care requisites, associated with life processes and the maintenance of the integrity of human structure and functioning; Developmental requisites, related to some natural condition of the life cycle or associated with some event; and Health Deviation requisites, related to disease conditions^10^. 

It should be emphasized that the Orem’s Theory is a valid reference in the systematization of nursing care, being used in studies with PLWHA^7^ ^-^ ^9^.

Thus, the use of Orem’s Theory is justified considering that self-care is indispensable to PLWHA in their daily life, with the intention of ensuring development for the benefit of life, health and well-being.

During the Nursing Consultation held with the PLWHA in a specialized outpatient clinic of infectology we realized the existence of aspects of the patients’ life that influenced in their self-care. However, it was not possible to quantify and verify statistically what these aspects were and whether they increased or decreased the self-care of PLWHA.

In view of this context, this study sought to analyze aspects that influence the increase or decrease of self-care of PLWHA treated in a specialized outpatient clinic.

## Method

This is a cross-sectional, analytical study, with quantitative approach, developed in a specialized outpatient clinic of infectology in a city in the interior of São Paulo, Brazil. The service is a reference for follow-up and treatment of cases of HIV and chronic hepatitis diagnosed in the region. Currently, the service serves approximately 800 PLWHA, which are divided into four weekly outpatient clinics, receiving multiprofessional care and demands in a spontaneous or referenced manner. 

The criterion that defined the patients for the study sample was the attendance on the days that the nursing consultation happens to the PLWHA. The non-probabilistic sampling totaled 135 PLWHA. However, in order to estimate the minimum differences that could be detected with n = 135 respondents, we considered simple random sampling, with type I error equal to 0.05 and type II error equal to 0.20, absence of confounding, standard deviation of the outcome equal to 2, “allocation” ratio equal to 1:1 for the binary independent variables and normality for the distribution of the outcome. With these assumptions, it was observed that the sample of 135 interviewees allowed detecting significant differences between two categories of a binary variable above a point. 

The nursing consultation has taken place in the specialized outpatient clinic of infectology since October 2013. It is developed by one of the authors and by students of the nursing undergraduate course who undergo training in this service, under the supervision of the researcher. In order to perform the nursing consultation, an instrument was constructed that had as theoretical reference the Orem’s Self-Care Theory. The construction of this instrument was based on authors who also perform the nursing consultation with PLWHA in other services, using instruments with the same theoretical framework^7^ ^-^ ^9^. 

The data collection was carried out between October 2014 and June 2017, in one of the outpatient clinics that take place on Wednesdays and has approximately 200 PlwHA enrolled. The data were collected on the same day of the nursing consultation. Four people refused to participate. In order to achieve the objective of the present study, it was necessary to elaborate an instrument with questions taken from a larger instrument, the nursing consultation, as quoted above. The consultations were carried out in a private environment, allowing the secrecy and confidentiality of the information obtained. It should be emphasized that in order to avoid repetition of the participants, all the PLWHA who perform the nursing consultation were registered in an Excel spreadsheet. Thus, before including a new participant, it was verified whether the participant was already part of the study.

The study included patients aged 18 years and older, of both sexes, with clinical and cognitive conditions to answer the study questions. 

The data collected were related to the independent variables and to the outcome of the study. The independent variables (sociodemographic and behavioral) obtained were sex, age, self-reported color, schooling, religion, partnership, number of children, family income, total dependents of income, residence with running water, sewage, garbage collection and proximity to health service, sexual orientation, sexual activity, time of knowledge of the diagnosis of HIV/AIDS infection, time of treatment and opportunistic diseases after diagnosis, good relationship with family, feeling of loneliness, sadness and/or distress, need for concealing the diagnosis, accepting the status of being HIV/AIDS, and having learned to live with HIV/AIDS. The selection of the independent variables occurred from research already developed with PLWHA^1^ ^,^ ^3^ ^,^ ^8^ ^-^ ^9^ ^,^ ^11^ ^-^ ^12^.

For the outcome, we considered the three self-care requisites: Universal, Health Deviation, and Developmental self-care requisites. The questions were constructed with binary response (yes or no). Each positive response to the development of self-care received one point. The higher the score, the higher the performance of self-care of PLWHA.

The Universal Self-Care Requisite was contemplated with 12 questions, namely: Do you receive guidance on nutrition? Do you follow the guidelines on nutrition? Do you drink at least eight glasses of water per day? Do you sleep at least eight hours a night? Do you perform physical exercises? Do you have some leisure activity and practice it weekly? Do you use some method in sexual intercourse to prevent reinfection of the virus? Do you participate in social activities? Do you often perform gynecological/urological examinations? Do you use drugs? Do you smoke? Do you drink alcohol? 

The questions of the Health Deviation Care Self-Care Requisite were composed of four questions, namely: Do they attend on the dates scheduled by the physician? Do you perform follow up with any other professionals when necessary? If you need to use a medicine (antiretroviral), do you use it daily? Do you go to the doctor only when you are sick? 

The Developmental Self-Care Requisite had the following question: Can you make changes in your lifestyle related to the disease? 

In the last three questions of Universal Self-Care and in the last question of Health Deviation Self-Care, we understood that the negative answer (no) would lead to the addition of a point and the positive answer (yes) would not result in a punctuation.

Facing the absence of a parameter to establish the aspects related to the increase or decrease of self-care in the PLWHA, the Universal Self-Care Score was elaborated consisting of the simple sum obtained from the answers given to 12 items that express universal self-care. Therefore, the score ranged from 0 to 12 points, and the higher the score, the greater the self-care. The Health Deviation Self-Care Score was also elaborated, ranging from 0 to 4 points. The higher the score, the greater the self-care. Developmental Self-care was considered positive if the study participant answered yes to the question: Can you make changes in your lifestyle related to the disease?

Statistical analyzes were performed using SPSS v21.0 software. The outcome of the Universal Self-Care Score was performed by a linear regression model with a normal response. The analysis of the chances of developmental and health deviation self-care was made by logistic regression models. Associations were considered statistically significant if p <0.05.

The study was approved by the Research Ethics Committee under protocol no. 563,918, in compliance with the recommendations of Resolution 466/2012 of the National Health Council. Participants signed the Informed Consent Form, thus guaranteeing anonymity.

## Results

Table 1 shows the independent (sociodemographic) variables of PLWHA. Considering the 135 people investigated, 56.3% were male, white, had children and religion. Age ranged from 19 to 74 years, with an average of 42.1 years (SD = 12). They had low levels of schooling (56.3%), 23% lived with up to one minimum wage and had, on average, 3.6 dependents of family income (SD = 1.8), minimum 1 and maximum 11. 

Few people had residence with garbage collection (3%), running water (4.5%) and sewage network (4.5%). However, 34.9% did not have a health service close to their home.

**Table 1:** Distribution of the independent (sociodemographic) variables of patients living with HIV/AIDS*. Botucatu, SP, Brazil, 2014-2017

**Variable**	**n**	**%**
**Sex**		
**Male**	**76**	**56.3**
**Female**	**59**	**43.7**
**Schooling**		
**Illiterate to incomplete primary education**	**43**	**31.9**
**Complete primary education to incomplete high school**	**33**	**24.4**
**Complete high school to incomplete higher education**	**49**	**36.3**
**Complete higher education**	**10**	**7.4**
**Self-referred color**		
**White**	**76**	**56.3**
**Non-white**	**59**	**43.7**
**Religion**		
**Yes**	**122**	**90.3**
**No**	**13**	**9.7**
**Has children**		
**Yes**	**83**	**61.4**
**No**	**52**	**38.6**
**Family income** ^**†**^		
**≤ 1**	**31**	**23.0**
**1.1 to 4**	**88**	**65.2**
**≥ 4.1**	**16**	**11.9**
**Number of family income dependents**		
**1 to 2**	**42**	**31.1**
**3 to 4**	**53**	**39.3**
**5 to 6**	**29**	**21.5**
**7 or more**	**11**	**8.1**
**Residence with garbage collection**		
**Yes**	**131**	**97.0**
**No**	**7**	**3.0**
**Residence with running water**		
**Yes**	**129**	**95.5**
**No**	**6**	**4.5**
**Residence with sewage network**		
**Yes**	**129**	**95.5**
**No**	**6**	**4.5**
**Health care next to residence**		
**Yes**	**88**	**65.1**
**No**	**47**	**34.9**

*Human Immunodeficiency Virus (HIV) and Human Immunodeficiency Syndrome (AIDS); ^†^Minimum wage in Brazil (2014 - R$ 724.00; 2015 - R$ 788.00; 2016 - R$ 880.00, 2017 - R$ 937.00)

Table 2 shows that most participants were heterosexual (75.6%), had a permanent partner (50.3%), active sexual life (54.8%), reported having a good relationship with the family after diagnosis of HIV infection (86.6%), accepted to have HIV/AIDS (77.1%), and had learned to live with the infection (87.4%).

However, 57.7% reported that after the diagnosis they had a change in their sexual life, felt loneliness, sadness and/or distress (51.1%), needed to hide the diagnosis (71.8%), and had contracted opportunistic diseases after the diagnosis (37.8%).

The mean time of diagnosis was 8.3 years (standard deviation (SD) = 7.1) and the mean duration of treatment was 7.3 years (SD = 6.7).

**Table 2:** Distribution of independent variables (behavior) of patients living with HIV/AIDS*. Botucatu, SP, Brazil, 2014-2017

**Variable**	**n**	**%**
**Sexual orientation**		
**Heterosexual**	**102**	**75.6**
**Homosexual**	**25**	**18.5**
**Bisexual**	**8**	**5.9**
**Has a permanent partner**		
**Yes**	**68**	**50.3**
**No**	**67**	**49.7**
**Active sexual life**		
**Yes**	**74**	**54.8**
**No**	**61**	**45.2**
**Alteration of sexual life after diagnosis**		
**Yes**	**78**	**57.7**
**No**	**57**	**42.3**
**Good relationship with family after diagnosis**		
**Yes**	**117**	**86.6**
**No**	**18**	**13.4**
**Feels loneliness, sadness and/or distress after the diagnosis**		
**Yes**	**69**	**51.1**
**No**	**66**	**48.9**
**Need to hide the diagnosis**		
**Yes**	**97**	**71.8**
**No**	**38**	**28.2**
Accepts to be with HIV/AIDS*		
**Yes**	**104**	**77.1**
**No**	**31**	**22.9**
Learned to live with HIV/AIDS*		
**Yes**	**118**	**87.4**
**No**	**17**	**12.6**
**Presented a change in lifestyle after diagnosis**		
**Yes**	**71**	**52.6**
**No**	**64**	**47.4**
**Opportunistic disease after diagnosis**		
**Yes**	**51**	**37.8**
**No**	**84**	**62.2**

*Human immunodeficiency virus (HIV) and Human Immunodeficiency Syndrome (AIDS)

The Self-Care Score was calculated considering 12 items and each item received the value of one point. The mean self-care score was 6.3 points (SD = 1.8), minimum of 1 point and maximum of 10 points. 

Table 3 shows the result of the analysis of this score with the intention to verify association with increase or decrease of self-care. At first, there was no statistically significant association. For this reason, we chose to select the independent variables that had lower p values and they were taken to a more parsimonious model.

In this second model, when evaluating the independent variables correlated with the Universal Self-Care Score, we observed that there was no positive correlation that evidences the increase in self-care. On the other hand, a statistically significant negative correlation was obtained (β = -0.72 (-1.38, -0.06), p <0.031) when it was evidenced that the PLWHA who needed to conceal the diagnosis performed less self-care.

**Table 3:** Linear regression model with normal response for the Universal Self-Care Score. Botucatu, SP, Brazil, 2014-2017

**Variable**	β* (95%CI) ^**†**^ **;** ***p*** ^**‡**^	**β** ^**§**^ **(95%CI)** ^**||**^ **;** ***p*** ^**¶**^
**Male sex**	**0.42 (-0.37,1.22),<0.293**	
**Age**	**0.01 (-0.01,0.03),<0.272**	
**Non-white**	**-0.27 (-0.88,0.34),<0.388**	
**Complete higher education**	**0.89 (-0.45,2.25),<0.194**	**0.45 (-0.76,1.67),<0.462**
**Complete high school to incomplete higher education**	**-0.11 (-0.89,0.67),<0.782**	**0.42 (-1.16,0.31),<0.257**
**Complete primary education to incomplete high school**	**-0.69 (-1.50,0.11),<0.093**	**-0.77 (-1.57,0.02),<0.058**
**Residence with garbage collection**	**-2.77 (-5.82,0.26),<0.074**	**-0.88 (-2.64,0.87),<0.323**
**Residence with running water**	**1.59 (-0.88,4.07),<0.207**	
**Health care next to residence**	**0.06 (-0.62,0.75),<0.854**	
**Family income ≥ 4.1 minimum wages**	**-0.19 (-1.36,0.97),<0.749**	
**Family income of 1.1 to 4 minimum wages**	**-0.32 (-1.10,0.44),<0.403**	
**Has religion**	**-0.36 (-1.48,0.76),<0.527**	
**With children**	**0.50 (-0.37,1.37),<0.261**	
**Number of dependents**	**0.04 (-0.12,0.20),<0.637**	
**Bisexual**	**-0.57 (-2.02,0.87),<0.440**	
**Homosexual**	**-0.46 (-1.54,0.61),<0.401**	
**With active sex life**	**0.25 (-0.45,0.95),<0.484**	
**With permanent partner**	**0.65 (-0.26,1.57),<0.161**	**0.58 (-0.02,1.18),<0.059**
**With change in sex life**	**0.05 (-0.58,0.69),<0.873**	
**Good family relationship after diagnosis**	**0.58 (-0.33,1.49),<0.212**	
**Feels loneliness, sadness and/or distress after the diagnosis**	**-0.23 (-0.89,0.42),<0.484**	
**Need to hide the diagnosis**	**-0.57 (-1.27,0.12),<0.109**	**-0.72 (-1.38 , -0.06),<0.031**
Accepts to be with HIV/AIDS**	**-0.07 (-0.89,0.75),<0.867**	
Learned to live with HIV/AIDS**	**0.52 (-0.47,1.51),<0.305**	
**Had opportunistic disease after diagnosis**	**-0.57 (-1.19,0.04),<0.071**	**-0.53 (-1.16,0.089),<0.093**
**Diagnostic time**	**0.01 (-0.07,0.10),<0.720**	
**Treatment time**	**-0.02 (-0.11,0.07),<0.692**	

*β - Beta; ^†^CI- Confidence Interval; ^‡^ *p*< 0,05; ^§^β - Beta (of the parsimonious model); ^||^CI- Confidence Interval (of the parsimonious model); ^¶^ *p*< 0.05 (of the parsimonious model); **Human immunodeficiency virus (HIV) and Human Immunodeficiency Syndrome (AIDS)

The Health Deviation Self-Care Score was established using four items and each item received the value of one point. The mean of the Health Deviation Score was 2.2 points (SD = 0.6), minimum of zero and maximum of four. Table 4 shows that there was need to take the independent variables that had a lower p-value to a more parsimonious model.

When the independent variables and the correlation with the Health Deviation Score were evaluated in this second model, we observed that the increase in the age of PLWHA decreases the chance of self-care (OR = 0.93 (0, 89, 0.97), p <0.003). However, PLWHA that have a permanent partner are more likely to perform self-care due to health deviation (OR = 3.46 (1.27, 9.46), p <0.015).

**Table 4:** Logistic regression models for the Health Deviation Self-Care Score. Botucatu, SP, Brazil, 2014-2017

**Variable**	OR*(95%CI) ^**†**^ **;** ***p*** ^**‡**^	**OR** ^**§**^ **(95%CI)** ^**||**^ **;** ***p*** ^**¶**^
**Male sex**	**0.20 (0.04,0.881),<0.033**	**0.40 (0.13,1.22),<0.110**
**Age**	**0.94 (0.89,0.99),<0.026**	**0.93 (0.89,0.97),<0.003**
**Non-white**	**0.62 (0.21,1.79),<0.385**	
**Illiterate to incomplete primary education**	**4.47 (1.08,18.45),<0.038**	**0.85 (0.52,1.39),<0.533**
**Complete primary education to incomplete high school**	**0.77 (0.20,2.95),<0.707**	
**Complete high school to incomplete higher education**	**0.52 (0.06,4.08),<0.538**	
**Family income ≤ 1 minimum wage**	**2.28 (0.66,7.90),<0.191**	**0.86 (0.40,1.83),<0.703**
**Family income of 1.1 to 4 minimum wages**	**0.78 (0.13,4.72),<0.794**	
**Has religion**	**0.09 (0.00,1.26),<0.075**	**0.21 (0.02,1.84),<0.161**
**With children**	**0.15 (0.02,0.82),<0.029**	**0.35 (0.10,1.22),<0.102**
**Number of dependents**	**0.85 (0.64,1.12),<0.261**	
**Heterosexual**	**1.13 (0.20,6.26),<0.888**	
**Homosexual**	**2.77 (0.14,53.93),<0.500**	
**With active sex life**	**1.09 (0.32,3.75),<0.881**	
**With permanent partner**	**3.83 (0.72,20.16),<0.113**	**3.46 (1.27,9.46),<0.015**
**With change in sex life**	**0.88 (0.29,2.70),<0.832**	
**Good family relationship after diagnosis**	**0.47 (0.09,2.30),<0.358**	
**Feels loneliness, sadness and/or distress after the diagnosis**	**0.57 (0.18,1.76),<0.329**	
**Need to hide the diagnosis**	**0.76 (0.23,2.45),<0.652**	
Accepts to be with HIV/AIDS**	**1.41 (0.34,5.79),<0.627**	
Learned to live with HIV/AIDS**	**0.43 (0.06,2.89),<0.386**	
**Had opportunistic disease after diagnosis**	**0.66 (0.22,1.96),<0.465**	
**Diagnostic time**	**1.05 (0.88,1.25),<0.555**	
**Treatment time**	**0.92 (0.76,1.11),<0.429**	

*OR - Odds ratio; ^†^CI- Confidence interval; ^‡^ *p*< 0.05; ^§^OR - Odds ratio (of the parsimonious model); ^||^CI- Confidence interval (of the parsimonious model); ^¶^ *p*< 0.05 (of the parsimonious model); **Human immunodeficiency virus (HIV) and Human Immunodeficiency Syndrome (AIDS)

Table 5 shows the association of the independent variables, such as the Developmental Self-Care Score, and it was considered positive if the PLWHA answered yes to the question: are you able to make changes in your lifestyle related to the disease? It was necessary to take the independent variables that had a lower p-value for a second more parsimonious model.

In this second model, it was observed that PLWHA that had a change in sexual life after HIV diagnosis has a greater chance of making changes in their lifestyle (OR = 1.94 (0.88, 4.28), p < 0.100). However, a spurious result was found when it was shown that PLWHA who had a good family relationship after diagnosis are less likely to make changes in their lifestyle (OR = 0.24 (0.06; 0.93); p <0.038).

**Table 5:** Logistic regression model for the Developmental Self-Care Score. Botucatu, SP, Brazil, 2014-2017

**Variable**	OR*(95%CI) ^**†**^ **;** ***p*** ^**‡**^	**OR** ^**§**^ **(95%CI)** ^**||**^ **;** ***p*** ^**¶**^
**Male sex**	**1.87 (0.58,6.00),<0.291**	
**Age**	**0.99 (0.96,1.02),<0.672**	
**Non-white**	**1.18 (0.49,2.80),<0.708**	
**Illiterate to incomplete primary education**	**1.40 (0.45,4.37),<0.558**	
**Complete primary education to incomplete high school**	**1.18 (0.39,3.56),<0.764**	
**Complete high school to incomplete higher education**	**3.23 (0.44,23.53),<0.246**	
**Family income ≤ 1 minimum wage**	**1.71 (0.58,5.07),<0.327**	
**Family income of 1.1 to 4 minimum wages**	**0.55 (0.10,3.03),<0.495**	
**Has religion**	**2.94 (0.57,15.10),<0.196**	**2.54 (0.66,9.73),<0.173**
**With children**	**2.77 (0.77,9.93),<0.118**	**1.09 (0.50,2.39),<0.828**
**Number of dependents**	**0.86 (0.67,1.10),<0.240**	
**Heterosexual**	**1.75 (0.38,7.97),<0.465**	
**Homosexual**	**2.13 (0.22,20.42),<0.511**	
**With active sex life**	**2.49 (0.87,7.09),<0.087**	**1.94 (0.88,4.28),<0.100**
**With permanent partner**	**0.78 (0.21,2.94),<0.724**	
**With change in sex life**	**2.43 (0.99,5.96),<0.053**	**2.79 (1.28,6.09),<0.010**
**Good family relationship after diagnosis**	**0.23 (0.05,1.08),<0.064**	**0.24 (0.06,0.93),<0.038**
**Feels loneliness, sadness and/or distress after the diagnosis**	**2.05 (0.80,5.22),<0.130**	**1.94 (0.87,4.34),<0.107**
**Need to hide the diagnosis**	**0.83 (0.31,2.24),<0.719**	
Accepts to be with HIV/AIDS**	**0.46 (0.13,1.52),<0.205**	
Learned to live with HIV/AIDS**	**1.58 (0.36,6.79),<0.537**	
**Had opportunistic disease after diagnosis**	**1.99 (0.82,4.82),<0.128**	**2.07 (0.94,4.57),<0.070**
**Diagnostic time**	**1.05 (0.92,1.19),<0.422**	
**Treatment time**	**0.92 (0.81,1.06),<0.269**	

*OR - Odds ratio; ^†^CI - Confidence interval; ^‡^ *p*< 0.05; ^§^OR - Odds ratio (of the parsimonious model); ^||^CI - Confidence interval (of the parsimonious model); ^¶^ *p*< 0.05 (of the parsimonious model); **Human immunodeficiency virus (HIV) and Human Immunodeficiency Syndrome (AIDS)

## Discussion

Since the beginning of the epidemic, 76.1 million people have been infected with HIV and 35 million people have died from AIDS-related causes. By 2016, there were 36.7 million PLWHA worldwide. The relevance of the AIDS epidemic can also be observed in the Brazilian context. From 1980 to June 2017, 882,810 AIDS cases were identified in the country. Annually, there have been an average of 40,000 new cases of this disease in the last five years^11^. 

Considering the comparison with other studies, the PLWHA profile of this study is in line with the latest Brazilian and world data, since the epidemic is concentrated in more vulnerable groups^11^ ^,^ ^13^.

The latest data from Brazil show that the epidemic is far from being controlled. From 1980 to June 2017, 576,245 (65.3%) cases of AIDS were recorded in men and 306,444 (34.7%) in women. The highest concentration of AIDS cases in Brazil is in individuals between the ages of 25 and 39, in both sexes. The main route of transmission between men and women 13 years of age or older was the sexual one. Among males, in the year 2016, the Southeast region presented a predominance of exposure of homosexuals (46.1% of cases), while in the other regions the predominance was of heterosexuals. When we analyze the AIDS cases in the last 10 years and the distribution of the individuals by race/color, we observed a decrease of 21.9% in the proportion of cases among white people. Among the self-reported brown people, the proportion increased 35.7%^11^. 

Similar to the data found in the present study, the highest concentration of AIDS cases occurred among individuals with incomplete secondary education (25.5%), although this range shows a tendency to reduce cases over the years. However, it is noted that men with AIDS had a higher education level than women. In 2016, the proportion of cases among illiterate men was 2.3%, while among women it was 3.9%. This fact was also observed in the complete higher education, in which among men the proportion was 13.1%, compared to 4.7% among women^11^.

Schooling reflects the economic situation of the people, which in turn plays an important role in the medical adherence of PLWHA. It is noteworthy that, after CD4 counting, adherence to antiretroviral therapy is the second largest predictor of progression to AIDS and death^3^. Adherence to antiretroviral therapy is known to be related to HIV suppression, decreased resistance rates, increased survival and improved quality of life^3^.

From the beginning of the AIDS epidemic to the end of 2016, 316,088 deaths were reported in Brazil, with HIV/AIDS as the main cause. However, in the period from 2014 to 2015, with the beginning of the policy of treatment for all, there was a reduction of 7.2% in the standardized mortality rate, which decreased from 5.7 to 5.3/100,000 inhabitants. In the period from 2006 to 2016, there was a decrease in the standardized mortality coefficient in Brazil, which went from 5.9 to 5.2 deaths per 100 thousand inhabitants, which corresponds to a reduction of 11.9%^11^.

Living with HIV/AIDS often brings a compromised functioning of the organism, causing a health deviation that requires the patient to be an active agent of self-care, since HIV leads to a chronic disease that until now has no cure. 

Studies have shown the importance of encouraging PLWHA to practice self-care, with the aim of contributing to the maintenance of their health^2^ ^,^ ^9^, leading the individual to understand that self-care is something that must be learned for their own benefit^14^. 

A study conducted in Iran has shown that providing adequate support and services, as well as a positive attitude of society towards HIV-positive women, can contribute to adherence to self-care in young women with HIV^1^. 

In contrast, HIV stigma can have numerous repercussions, such as loss of friendships and family ties, dismissal from school and occupation, and denial of health care^15^ ^-^ ^16^. HIV stigma is a process of devaluation of people living with or who are associated with HIV infection and may be related to the non-disclosure of their HIV status^4^. 

As found in the present study, concealing the diagnosis leads PLWHA to perform less self-care. This fact can be justified by the stigma of HIV that leads many PLWHA not to seek a health service to perform the treatment^17^. In the world, due to the stigma, one third of PLWHA do not reveal their positive serology for HIV to others^15^.

It was also demonstrated in this study that age was a variable that negatively influenced the performance of self-care. Study shows that elderly people living with HIV/AIDS for almost 30 years and are part of the pre-antiretroviral therapy group have developed their own strategies for increasing resilience, including self-care behaviors, such as dedication to health and involvement in medical care. In contrast, the group of elderly people living in the post-antiretroviral therapy era understood self-care as remaining adherent to the therapy and thus abstained from good health behaviors^15^.

However, considering that adherence to antiretroviral therapy is a part of actions that demonstrate self-care, a study in Tanzania found that non-adherence to antiretroviral therapy was associated with younger age and unemployment^12^.

The present study found that having a permanent partner increases the chance for PLWHA to perform self-care. In this sense, we can see the scarcity of literature that specifically discusses the role of permanent partnership in the performance of self-care. Another finding was that PLWHA who maintained a good family relationship after diagnosis are less likely to make changes in their lifestyle. This data was considered as a spurious regression, since there is no relation of cause and effect.

However, a literature review on social networks to support PLWHA suggests that there is little scientific production on families in the context of HIV/AIDS. The authors emphasize that, although there are some difficulties in accessing family members, such as prejudice and fear of stigma, it is crucial that studies that favor family members and caregivers of PLWHA be developed^18^.

After the logistic regression, the present study revealed that PLWHA who present a change in sexual life after HIV diagnosis has a greater chance of making changes in their lifestyle. However, a study shows that HIV diagnosis leads PLWHA to have sexual dissatisfaction^19^. The persistence of HIV-related stigma and discrimination may be a barrier to the sexual life of PLWHA, which has a greater effect on the desire and frequency of sexual activity than on the discontinuation thereof, a fact influenced by HIV/AIDS stigma, and moral and religious values^20^.

However, it is believed that knowing aspects that may influence the performance of self-care contributes to the performance of the professionals who assist PLWHA. The importance of self-care measuring instruments is emphasized and can be used as a methodological tool that assists in the evaluation of patients’ responses to the performance of their self-care. 

The study has as limitations the fact that the participants were recruited in an specialized outpatient service, which tends to present samples of PLWHA that already perform better their self-care, since the recruitment occurred during the patients’ attendance to nursing consultations, which demonstrates that these patients care about their health. Therefore, the presence of the patient in the follow-up visit shows a greater performance of self-care by these subjects. The results should not be generalized to other populations and regions of the country.

## Conclusion

This study evidenced aspects related to the increase or decrease of self-care in PLWHA, which are attended in a specialized outpatient service. Among these aspects, it should be noted that PLWHA who needed to hide the diagnosis of HIV/AIDS had less self-care. The chance of self-care decreased with increasing age. On the other hand, it was found that PLWHA that have a permanent partner have a greater chance of performing self-care. 

The use of Orem’s Theory made it possible to identify the increase or decrease of self-care of PLWHA. However, other studies are necessary to emphasize the analytical character of the self-care performance of these patients.
